# AXL–GAS6 expression can predict for adverse prognosis in non-small cell lung cancer with brain metastases

**DOI:** 10.1007/s00432-017-2408-4

**Published:** 2017-05-27

**Authors:** Xiaoliang Wu, Wenjuan Ma, Qianghua Zhou, Haijuan Yan, Zuan-Fu Lim, Mayan Huang, Chuangzhong Deng, Xingsu Yu, Huifang Su, Satoshi Komo, Haixia Yang, Xinke Zhang, Sijin Wen, Zhenfeng Zhang, Patrick C. Ma

**Affiliations:** 1 State Key Laboratory of Oncology in South China and Collaborative Innovation Center for Cancer Medicine, Sun Yat-sen University Cancer Center, 651 Dongfeng East Rd, Guangzhou, 510060 China; 2 Department of Oncology, Guizhou Provincial People’s Hospital, Guiyang, China; 3 WVU Cancer Institute, Mary Babb Randolph Cancer Center, West Virginia University, Morgantown, WV USA; 4 Department of Biostatistics, West Virginia University, Morgantown, WV USA; 5 West Virginia Clinical and Translational Science Institute, Morgantown, WV USA; 6 Present Address: Department of Radiology, The Second Affiliated Hospital of Guangzhou Medical University, 250 Changgang Road, Guangzhou, 510260 China; 7 Department of Medical Imaging, Sun Yat-Sen University Cancer Center, 651 Dongfeng East Rd, Guangzhou, 510060 China; 8 Sara Crile Allen and James Frederick Allen Comprehensive Lung Cancer Program, Eminent Scholar in Lung Cancer Research, WVU Cancer Institute, West Virginia University, 1 Medical Center Drive, P.O. Box 9300, Morgantown, WV 26506-9300 USA

**Keywords:** NSCLC, Brain metastasis, AXL, GAS6, EMT, Survival, Prognostic biomarker

## Abstract

**Purpose:**

Patients with non-small cell lung cancer (NSCLC) brain metastases (BM) have poor clinical outcomes. We sought to determine if AXL–GAS6 expression can be used as independent prognostic biomarkers for NSCLC BM.

**Methods:**

We retrospectively studied the medical records of 98 patients diagnosed with advanced metastatic NSCLC from December 2000 to June 2014. Out of a total of 98 patients with NSCLC metastases, 66 patients were identified to have brain metastases. The expressions of AXL and GAS6 were assessed by standard immunohistochemistry and correlated with clinicopathological factors and overall survival (OS) outcomes.

**Results:**

The expression of AXL was positively associated with GAS6 expression (*P* < 0.001), and tumor differentiation (*P* = 0.014) in advanced NSCLC with metastases. AXL expression displayed no association with gender, age, smoking history, pathology, T stage, N stage, CEA, and LDH. In univariate analysis, both AXL and GAS6 were found to predict worse OS outcomes (AXL: HR 1.77, 95% CI 1.13–2.79, *P* = 0.01; GAS6: HR 1.80, 95% CI 1.14–2.84, *P* = 0.01). In the brain metastasis subgroup, the expression of AXL was positively associated with GAS6 expression (*P* < 0.001). Both AXL and GAS6 were found to predict worse BM-OS outcomes in univariate analysis (AXL: HR 2.19, 95% CI 1.33–4.10, *P* = 0.005; GAS6: HR 2.04, 95% CI 1.01–3.71, *P* = 0.019). In multivariate analysis, high co-expression of AXL/GAS6 was found to be an independent unfavorable risk factor for the overall study population (HR 2.33, 95% CI 1.40–3.87, *P* = 0.0011) and also in BM (HR 2.76, 95% CI 1.45–5.25, *P* = 0.001), predicting worse survival outcome.

**Conclusions:**

AXL–GAS6 co-expression represents a potential independent prognostic biomarker for survival outcome in NSCLC BM patients.

**Electronic supplementary material:**

The online version of this article (doi:10.1007/s00432-017-2408-4) contains supplementary material, which is available to authorized users.

## Introduction

Lung cancer is a disease with high morbidity and is the leading cause of cancer mortality worldwide (Chen et al. 2016; Siegel et al. 2016). Non-small-cell lung cancer (NSCLC) accounts for approximately 85% of all lung cancer cases (Ettinger et al. 2012). Unfortunately, although lung cancer treatments have had considerable advances over recent years, most of the cases present at late stages and remain incurable. Prognosis of patients with advanced NSCLC remains very poor, especially in patients with NSCLC brain metastases (BM). Among all patients with NSCLC, nearly 20–40% will develop brain metastases during the course of disease and ultimately succumb to it (Barnholtz-Sloan et al. 2004; Mujoomdar et al. 2007). The median survival time of patients with NSCLC BM is approximately 9.3–19.1 months (Baek et al. 2016; Sperduto et al. 2010; Welsh et al. 2013). Hence, it is an unmet need to identify potential biomarkers in NSCLC patients with BM to provide better insights into the disease prognostication. Also, it is crucial to identify novel potential actionable targets in this disease group that can be translated into therapeutic benefits to impact the clinical outcome.

AXL (also known as tyrosine-protein kinase receptor UFO, TYRO7, and ARK) is a receptor tyrosine kinase belonging to the TAM (TYRO3/AXL/MER) family (O’Bryan et al. 1991). AXL has been reported to be overexpressed or ectopically expressed in a multitude of human cancers, including breast (Zhang et al. 2008), colon (Craven et al. 1995), lung (Ishikawa et al. 2013), hepatocellular carcinoma (He et al. 2010), and esophageal adenocarcinoma (Hector et al. 2010). Its natural ligand, growth arrest-specific gene 6 (GAS6), can bind to AXL receptor kinase to activate its catalytic kinase activity, which in turn leads to downstream activation of various oncogenic signaling pathways, regulating cell proliferation, invasion, survival, metastases, anti-apoptosis, and drug resistance (Linger et al. 2008). These biological effects are mainly mediated by GAS6/AXL-induced activation of MAPK/ERK and PI3K/AKT signaling pathways (Hasanbasic et al. 2004; Hong et al. 2008; Lee et al. 2002).

Overexpression of AXL has been observed in nearly 60% of NSCLC cell lines, and it is also found highly expressed among primary lung cancers (Shieh et al. 2005; Wimmel et al. 2001). AXL has been nominated as a potent epithelial–mesenchymal transition (EMT) inducer, and a potential molecular target for lung cancer therapy (Shieh et al. 2005; Vaughan et al. 2012). Genetic knockdown of AXL using siRNA can inhibit invasion of NSCLC cells. Importantly, induced AXL–GAS6 signaling has also been reported to mediate cancer drug resistance against both cytotoxic chemotherapeutics and targeted therapies in lung cancer (Zhang et al. 2012). Although the role of AXL–GAS6 expression and signaling effects in lung cancer have been reported in recent years (Ishikawa et al. 2013; Shieh et al. 2005; Wimmel et al. 2001; Zhang et al. 2012), the role of AXL–GAS6 in brain metastases from lung cancer and its potential prognostic importance have not been well clarified.

In this study, we conducted a retrospective analysis to evaluate AXL and GAS6 expression levels in NSCLC patients with BM by standard immunohistochemistry (IHC) and further interrogated its potential role in survival outcome prognosis.

## Materials and methods

### Patient characteristics

All tissues were collected from 98 patients who had received surgical resection from December 2000 to June 2014 at the Department of Surgery of Sun Yat-sen University (Guangzhou, China). Ninety one (91) patients had distant metastases at the time of diagnosis. The remaining seven patients were without metastases at initial diagnosis, but continued monitoring revealed that there was eventually metastatic disease. A subgroup of 66 patients had distant metastases to the brain, while the other 32 patients had distant metastases to other organs (liver, bone, or adrenal gland). The eligibility criteria are as follows: (1) All the patients had developed distant metastases. (2) All the pathological diagnoses of tumor tissues were confirmed to be NSCLC by anatomic pathologist. The cases were selected consecutively on the basis of availability of resection tissues and follow-up data. (3) Tumor stage was classified according to the 7th lung cancer TNM staging edition from International Association for the Study of Lung Cancer (IASLC).

### Immunonhistochemical (IHC) staining

A total of 98 tumor tissue samples [51 samples from lung, 45 samples from other organs: brain (*n* = 35), adrenal gland (*n* = 5), kidney (*n* = 2), liver (*n* = 2), bone (*n* = 1)] were used in the IHC analysis. Formalin-fixed, paraffin-embedded tumor specimens were cut into 5-µm sections. The samples were deparaffinized in xylene and rehydrated in a series of graded ethanol after being baked at 60 °C for 2 h. Next, the samples were incubated with 3% hydrogen peroxide for 15 min to block endogenous peroxidase activity. The sections were microwaved in 1 mM ethylenediamine tetra-acetic acid (EDTA, pH 8.0) for 25 min for antigen retrieval, and then pre-incubated in 2.5% normal horse serum (VECTOR, #S-2012) for 30 min to block non-specific staining. The sections were incubated with the primary rabbit anti-human AXL monoclonal antibody (working dilution 1:200, Cell Signaling Technology, #8661) and goat anti-human GAS6 polyclonal antibody (working dilution 1:200, R&D Systems, #AF885-SP) overnight at 4 °C. Subsequently, the samples were incubated with secondary antibody (VECTOR, #MP-7500, #MP-7405) at room temperature for 1 h. All specimens were scored independently by two experienced pathologists who were blinded to the patients’ identity and clinical status. H-scores of percentage of positive tumor cells (0–100%) and dominant staining intensity (0, 1+, 2+, and 3+) of immunostaining were adopted for the expression data analysis. The final quantitation of each staining was determined by averaging the *H*-scores (% positive tumor cells × staining intensity) obtained from the two pathologists. AXL expression was classified as high or low based on whether the *H*-score was above or below the median cut-off score of 0.4, respectively. GAS6 expression was considered high if the score was above 0.3 as the median cut-off.

### Follow-up

The last date of follow-up was on June 30, 2016. In all patients (47 females and 51 males), the follow-up period ranged from 3 to 67 months. All patients were followed up every 3 months in the first year and every 6 months thereafter. The follow-up protocol included physical examination, carcinoembryonic antigen (CEA) level, brain MRI, chest X-ray or CT, and abdominal ultrasonography. During the course of follow-up, 79 patients (80.6%) died of lung cancer-related causes. 19 patients were still alive at the time of the last follow-up report.

### Statistical analysis

Overall survival (OS) was defined as the time from diagnosis of metastatic NSCLC to the death of the patient or last date of follow-up. Overall survival of the brain metastatic NSCLC subgroup (BM-OS) was calculated from the time of diagnosis of the first BM to the time of death of the patient or last date of follow-up. The SPSS software package (version 24.0, IBM, USA) and GraphPad Prism (version 6.0, GraphPad Software Inc, USA) were used for the statistical analysis. The Chi-square test was used to assess the correlation of AXL–GAS6 status with clinicopathological characteristics. Survival curves were generated using the Kaplan–Meier method, and differences between curves were assessed by the log-rank test. The Cox multivariate proportional hazards regression model was used to determine the independent risk factors that influence overall survival. *P* values less than 0.05 were considered to be statistically significant.

## Results

### AXL and GAS6 expression in NSCLC

**Fig. 1:**
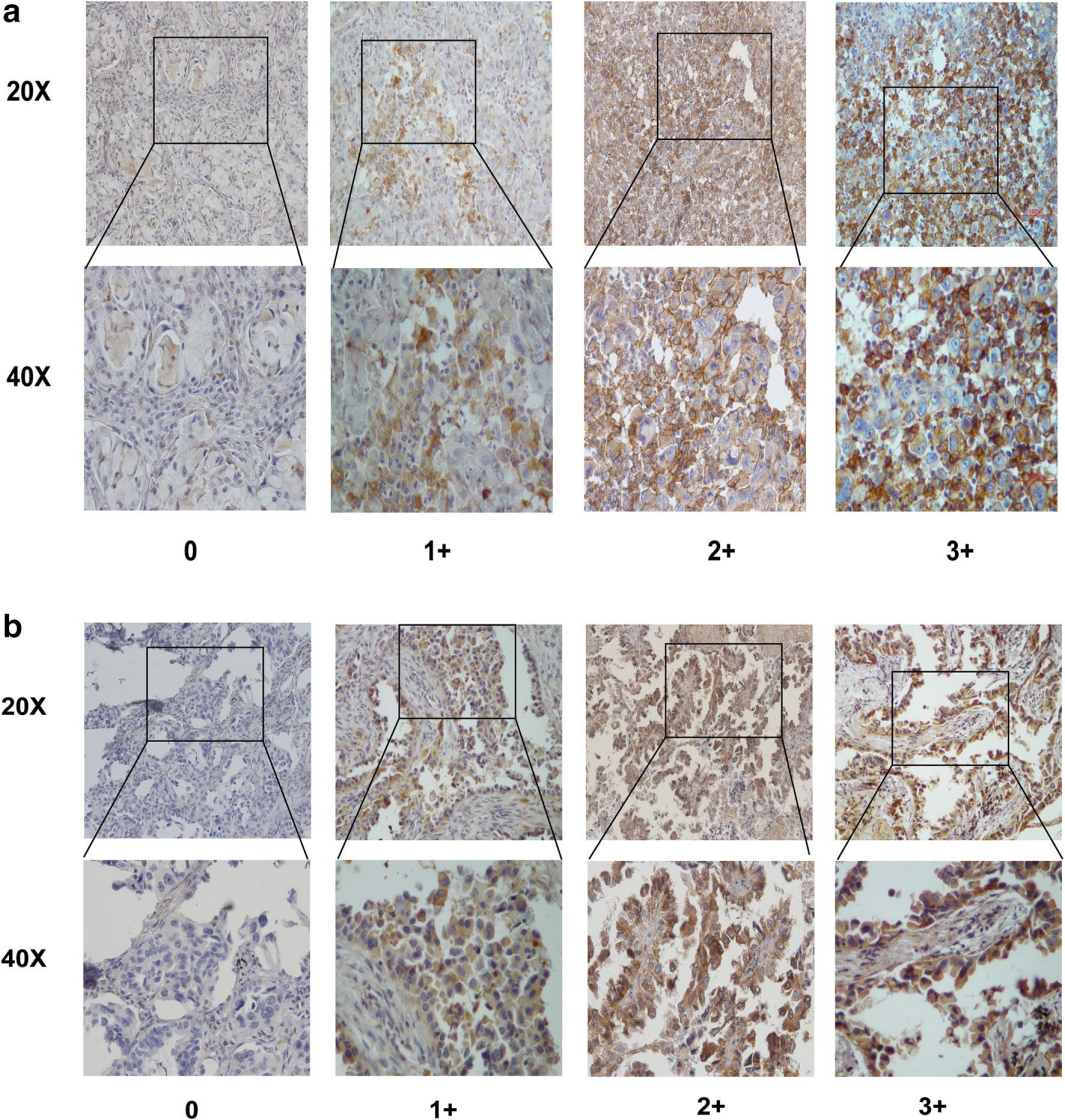
AXL and GAS6 expression in NSCLC tumor tissues. **a** Examples of tumoral staining intensity (0, 1+, 2+, and 3+) of AXL in immunohistochemistry (IHC) analysis. **b** Examples of tumoral staining intensity (0, 1+, 2+, and 3+) of GAS6 in immunohistochemistry (IHC) analysis

To elucidate the biological significance of AXL in lung cancer metastases, especially in NSCLC with brain metastases, we tested the expression of AXL and its ligand GAS6 in the selected 98 lung cancer specimens by immunohistochemical staining. The results showed that AXL and GAS6 expressions are primarily localized both at the cytoplasmic membrane and within the cytoplasm of tumor cells, respectively (Fig. 1). In the brain metastasis subgroup, high AXL and GAS6 expression was found in 36 of 66 (54.5%) and 37 of 66 (56.0%) patients, respectively. In the subgroup of lung cancer with metastases to organs other than brain, high AXL and GAS6 expression accounted for nine of 32 (28.1%) in both markers.

### Correlation of AXL–GAS6 protein expression with clinicopathological parameters in lung cancer with metastases

To gain insight into the potential role of AXL in NSCLC with metastases, we correlated AXL expression status in the study cohort of 98 NSCLC patients with distant metastases with various clinical and pathological features. The expression of AXL was significantly correlated with tumor differentiation (*P* = 0.014). The association between AXL and GAS6 expressions was estimated using Spearman’s correlation with the scatterplot and fitted straight line and was found to have a positive correlation (*R* = 0.532, *P* < 0.001) (Figure S1). In contrast, AXL expression displayed no association with gender, age, smoking history, pathology, T stage, N stage, CEA, or LDH (all *P* > 0.05) (Table 1).

**Table 1:** Correlation of AXL expression with clinicopathological parameters in lung cancer metastases

Characteristics	Total	Low AXL (%)	High AXL (%)	*P* value
Gender				0.56
Male	51	22 (43.1)	29 (56.9)	
Female	47	27 (57.4)	20 (42.6)	
Age, years				0.39
≤50	34	15 (44.1)	19 (55.9)	
>50	64	34 (53.1)	30 (46.9)	
Smoking history				0.47
Yes	40	18 (45)	22 (55)	
No	58	31 (53.4)	27 (46.6)	
Pathology				0.18
Adenocarcinoma	88	46 (52.3)	42 (47.7)	
Non-adeno	10	3 (30)	7 (70)	
Tumor differentiation				0.014
Poor	53	20 (37.7)	33 (62.3)	
Moderate and well	45	29 (64.4)	16 (35.6)	
T stage				0.32
1/2	77	37 (48.1)	40 (51.9)	
3/4	21	14 (66.7)	7 (33.3)	
N stage				0.54
0/1	40	23 (57.5)	17 (42.5)	
2/3	58	27 (46.5)	31 (53.5)	
CEA (ng/mL)				0.22
≤5	46	20 (43.5)	26 (56.5)	
>5	52	29 (55.8)	23 (44.2)	
LDH (u/L)				0.79
≤245	81	41 (50.6)	40 (49.4)	
>245	17	8 (47.1)	9 (52.9)	
GAS6 expression				<0.001
Low	49	37 (75.5)	12 (24.5)	
High	49	12 (24.5)	37 (75.5)	

**Fig. 2:**
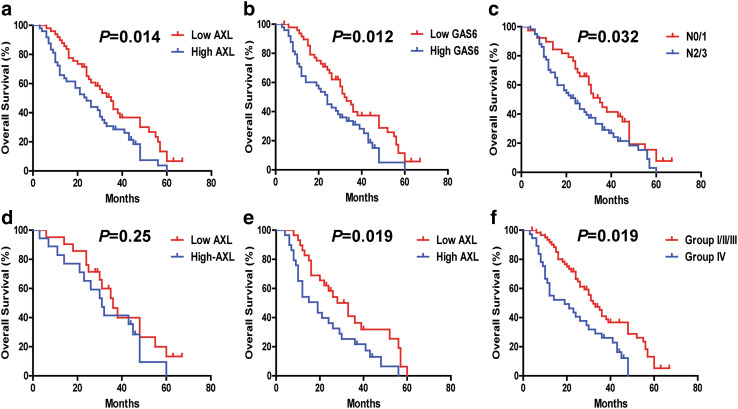
The correlation of NSCLC overall survival (OS) with different clinicopathological characteristics. **a**–**c** Survival curves were generated using the Kaplan–Meier method, and differences between survival curves were estimated by the log-rank test. AXL, GAS6, and nodal (N) stage have statistically significant correlation with OS differences. **d, e** The correlation between AXL expression and NSCLC OS in nodal (N) stage group. **d** N 0/1 stage subgroup (*n* = 40, *P* > 0.05). **e** N 2/3 stage subgroup (*n* = 58, *P* < 0.05). **f** Combined analysis of AXL and GAS6 co-expression and its correlation with NSCLC metastasis overall survival. The association of AXL/GAS6 co-expression with overall survival (log-rank *P* < 0.05) is shown here. Group I with AXL^Low^ and GAS6^Low^ (*n* = 37); Group II with AXL^High^ and GAS6^Low^ (*n* = 12); Group III with AXL^Low^ and GAS6^High^ (*n* = 12); Group IV, AXL^High^ and GAS6^High^ (*n* = 37)

We next analyzed the correlation between AXL and traditional clinicopathological parameters with patients’ outcomes by univariate analysis. Significantly increased OS was observed for the NSCLC metastasis-positive patients with low AXL expression (AXL^Low^) (*P* = 0.014), low GAS6 (GAS6^Low^) (*P* = 0.012), N 0/1 (*P* = 0.032) (Fig. 2a–c), but not with other clinicopathological parameters (Figure S2, a–h). In addition, the median OS in the group with low levels of AXL (AXL^Low^) (*n* = 49) and in the group with high levels of AXL (AXL^High^) (*n* = 49) were 35 and 24 months, respectively. Furthermore, the 2-year OS rates of AXL^Low^ vs AXL^High^ were 65.1 and 48.3%, respectively (Table 2).

**Table 2:** Two-year overall survival (OS) rates of AXL^Low^ and AXL^High^ tumors in NSCLC metastasis and NSCLC BM groups

	NSCLC metastases	NSCLC brain metastases
Median OS (months)		
Low AXL (mo)	35	48
High AXL (mo)	24	21
2-year survival rates		
Low AXL (%)	65.1	68.8
High AXL (%)	48.3	44.8

We also performed univariate and multivariate Cox model analyses to analyze whether AXL–GAS6 represent potential independent predictors for OS outcome in patients with NSCLC metastases. Both AXL and GAS6 were found to predict OS outcomes in our univariate analysis (AXL: HR 1.77, 95% CI 1.13–2.79, *P* = 0.01; GAS6: HR 1.80, 95% CI 1.14–2.84, *P* = 0.01) (Table 3). Combined AXL/GAS6 co-expression analysis yielded stronger predictor index with HR 2.20 (95% CI 1.36–3.56, *P* = 0.001) in the AXL^High^/GAS6^High^ group. Multivariate analysis of AXL/GAS6 co-expression significantly correlated with OS (HR 2.33, 95% CI 1.40–3.87, *P* = 0.0011) (Table 4). Our results revealed that AXL/GAS6 co-expression and N stage (HR 1.81, 95% CI 1.13–2.90, *P* = 0.013) were independent poor predictors for OS in patients with NSCLC metastases (Tables 3, 4).

**Table 3:** Univariate analysis of the correlation of AXL with overall survival in patients with NSCLC metastasis

	HR	95% CI	*P* value
Gender (male vs female)	0.93	0.60–1.47	0.78
Age, years (≤50 vs >50)	1.08	0.68–1.74	0.76
Smoking history (yes or no)	1.18	0.78–1.78	0.44
Pathology (adeno vs non-adeno)	1.00	0.48–2.08	1.00
Tumor differentiation (poor vs moderate and well)	1.07	0.69–1.67	0.77
T stage (T 3/4 vs T 1/2)	1.23	0.46–1.42	0.44
LDH (u/L) (≤245 vs >245)	1.27	0.72–2.23	0.41
CEA (ng/mL) (≤5 vs >5)	1.53	0.98–2.41	0.07
N stage (N 0/1 vs N 2/3)	1.65	1.04–2.61	0.03
AXL (high vs low)	1.77	1.13–2.79	0.01
GAS6 (high vs low)	1.80	1.14–2.84	0.01
AXL/GAS6 (IV vs I/II/III)	2.20	1.36–3.56	0.001

**Table 4:** Multivariate analysis of the correlation of AXL with overall survival in patients with NSCLC metastases

	HR	95% CI	*P* value
N stage (N 2/3 vs N 0/1)	1.81	1.13–2.90	0.013
AXL/GAS6 (IV vs I/II/III)	2.33	1.40–3.87	0.0011

We also sought to determine whether the prognostic value of AXL changes among different N stages. The AXL^High^ expression status maintained its prognostic value in predicting shorter OS in N 2/3 stage (*n* = 58) (*P* = 0.019), but not in N 0/1 stage (*n* = 40) (*P* = 0.25) (Fig. 2d, e).

### High expression of AXL–GAS6 is significantly associated with poor prognosis in patients with NSCLC with BM

In the subgroup of patients with NSCLC with brain metastases, the high expression of AXL (AXL^High^) was significantly associated with GAS6 expression (*P* < 0.001), but not with tumor differentiation, gender, age, smoking history, pathology, T stage, N stage, CEA, and LDH (all *P* > 0.05) (Table 5).

**Table 5:** Correlation of AXL expression with clinicopathological parameters in NSCLC brain metastases

Characteristics	Total	Low AXL (%)	High AXL (%)	*P* value
Gender				0.21
Male	34	18 (58.9)	16 (41.1)	
Female	32	12 (43.8)	20 (56.2)	
Age (years)				0.89
≤50	27	12 (48.2)	15 (51.8)	
>50	39	18 (51.2)	21 (48.8)	
Smoking history				0.59
Yes	28	14 (53.6)	14 (46.4)	
No	38	16 (47.4)	22 (52.6)	
Pathology				0.85
Adenocarcinoma	62	28 (52.3)	34 (47.7)	
Non-adeno	4	2 (30)	2 (70)	
Tumor differentiation				0.83
Poor	35	13 (42.8)	22 (57.2)	
Moderate and well	31	17 (58.1)	14 (41.9)	
T stage				0.49
1/2	51	22 (45.1)	29 (54.9)	
3/4	15	8 (66.7)	7 (33.3)	
N stage				0.19
0/1	23	14 (60.9)	9 (39.1)	
2/3	43	19 (44.2)	24 (55.8)	
CEA(ng/mL)				0.087
≤5	34	12 (41.2)	22 (58.8)	
>5	32	18 (59.4)	14 (40.6)	
LDH(u/L)				0.74
≤245	55	27 (49.1)	28 (50.9)	
>245	11	6 (54.6)	5 (45.4)	
GAS6 expression				<0.001
Low	29	20 (68.9)	9 (31.1)	
High	37	9 (24.4)	28 (75.6)	
Tissue				0.49
Lung	31	15 (54.9)	16 (45.1)	
Brain	35	14 (45.8)	21 (54.2)	

		Low AXL/GAS6 (%)	High AXL/GAS6 (%)	
Tissue				
Lung	21	12 (57.2)	9 (42.8)	0.021
Brain	26	7 (26.9)	19 (73.1)	

**Fig. 3:**
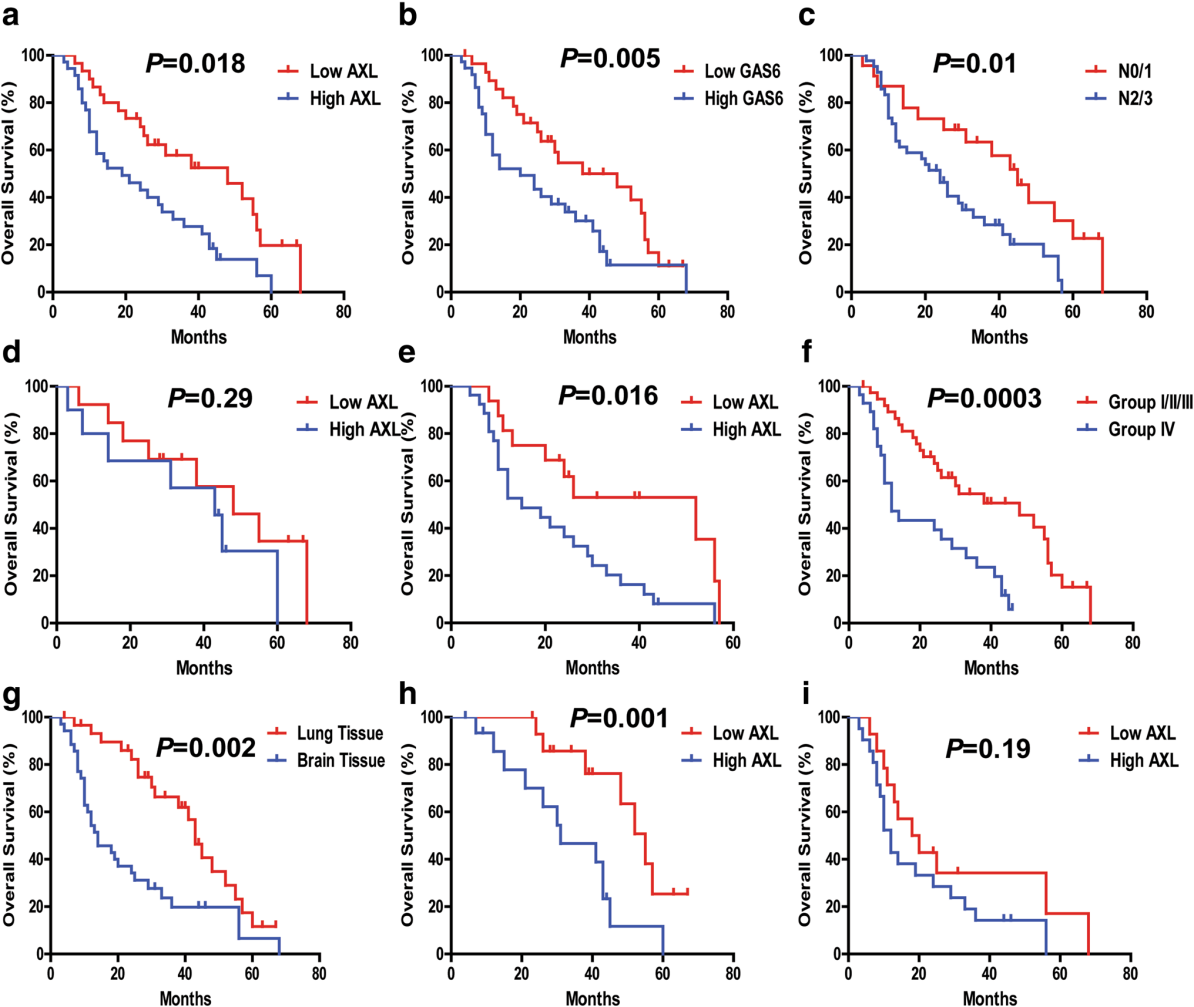
The correlation of NSCLC BM-OS with different clinicopathological characteristics. **a**–**c** AXL, GAS6, and nodal (N) stage have statistically significant correlation with BM-OS. **d, e** The correlation between AXL expression and NSCLC BM-OS in N 2/3 stage group (*n* = 43), *P* < 0.05. **f** Combined analysis of AXL and GAS6 co-expression and its correlation with NSCLC BM-OS. The association of AXL/GAS6 high co-expression with overall survival (log-rank *P* < 0.05) is shown here. Group I with AXL^Low^ and GAS6^Low^ (*n* = 20); Group II with AXL^High^ and GAS6^Low^ (*n* = 9); Group III with AXL^Low^ and GAS6^High^ (*n* = 9); Group IV, AXL^High^ and GAS6^High^ (n = 26). **g**–**i** The correlation between AXL expression and NSCLC BM-OS in the lung tissue subgroup and brain tissue subgroup. AXL expression carries a statistically significant BM-OS difference in lung tissue subgroup, and not in brain tissue subgroup

Significant OS disadvantages were observed for the NSCLC metastasis patients with high AXL expression (AXL^High^), high GAS6 expression (GAS6^High^), and N 2/3 stage (*P* < 0.01), compared with the AXL^Low^, GAS6^Low^, N 0/1 stage group, respectively (Fig. 3a–c). Median OS for AXL^Low^ (*n* = 29) and AXL^High^ (*n* = 37) were 48 and 21 months, respectively (*P* < 0.05). Furthermore, the 2-year OS rates of the AXL^low^ (*n* = 29) and AXL^high^ were 68.8% and 44.8%, respectively (Table 2).

We used both univariate and multivariate Cox models to analyze whether AXL–GAS6 could be an independent predictor for BM-OS in patients with NSCLC with brain metastases. Our results in the univariate analysis revealed that high expression of AXL (AXL^High^) (HR 2.19, 95% CI 1.33–4.10, *P* = 0.005), and GAS6 (GAS6^High^) (HR 2.04, 95% CI 1.01–3.71, *P* = 0.019), and N stage (HR 2.08, 95% CI 1.24–3.82, *P* = 0.01) were predictors for poor OS in patients with NSCLC with BM (Table 6). In the multivariate Cox analysis model, combined AXL/GAS6 co-expression significantly predicts poor OS with HR 2.76 (95% CI 1.45–5.25, *P* = 0.001) in the AXL^High^/GAS6^High^ group (Group IV vs I/II/III) (Table 7).

**Table 6:** Univariate analysis of the correlation of AXL with overall survival in patients with NSCLC brain metastases

	HR	95% CI	*P* value
Gender (male vs female)	1.39	0.78–2.50	0.26
Age, years (>50 vs ≤50)	1.21	0.46–1.51	0.54
Smoking history (yes vs no)	1.46	0.81–2.66	0.20
Tumor differentiation (poor vs moderate and well)	1.37	0.77–2.49	0.28
T stage (T3/4 vs T1/2)	1.02	0.53–1.96	0.95
LDH (u/L) (>245 vs ≤245)	1.26	0.57–2.78	0.56
CEA (ng/mL) (>5 vs ≤5)	1.32	0.74–2.35	0.34
N stage (N2/3 vs N0/1)	2.08	1.24–3.82	0.01
AXL (high vs low)	2.19	1.33–4.10	0.005
GAS6 (high vs low)	2.04	1.01–3.71	0.019
AXL/GAS6 (IV vs I/II/III)	2.44	1.44–4.17	0.001

**Table 7:** Multivariate analysis of the correlation of AXL with overall survival in patients with NSCLC brain metastases

	HR	95% CI	*P* value
N stage (N2/3 vs N0/1)	2.12	1.08–4.17	0.029
AXL/GAS6 (IV vs I/II/III)	2.76	1.45–5.25	0.001

We further sought to determine whether the prognostic value of AXL changes in different N stages of NSCLC BM patients. The high expression of AXL (AXL^High^) maintained its prognostic value in predicting shorter BM-OS in N 2/3 stage (*P* = 0.016) (Fig. 3e), not in N 0/1 stage (*P* = 0.29) (Fig. 3d).

Significant OS advantages were observed for the NSCLC BM in lung tissue subgroup compared with the brain tissue subgroup. The AXL^High^ expression has prognostic value in predicting shorter OS in lung tissue group (*n* = 31) (*P* = 0.001), but not significantly so in the brain tissue group (*n* = 35) (*P* = 0.19) (Fig. 3g–i). In addition, the brain tissues comparing with the lung tissues was associated with significantly higher co-expression of AXL/GAS6 (AXL^High^/GAS6^High^) (*P* = 0.021), albeit not with just high expression of AXL alone (*P* = 0.49) (Table 5).

In the patient subgroup with NSCLC with other organ metastases (OM), the high expression of AXL/GAS6/N stage was not associated with OS (*P* > 0.05) (Figure S3, a–c).

### Combination of AXL and GAS6 co-expression is associated with poor prognosis in advanced NSCLC with distant metastases especially in the BM group

We found that 37/49 (75.5%) patients with GAS6^Low^ expression also had AXL^Low^ expression (i.e., GAS6^Low^/AXL^Low^), while 75.5% (37/49) of GAS6^High^ patient also had AXL^High^ co-expression (GAS6^High^/AXL^High^) (Table 1). In the patient subgroup with NSCLC with brain metastases, 20/29 (68.9%) of GAS6^Low^ patients were found also to have AXL^Low^ expression (GAS6^Low^/AXL^Low^), while 28/37 (75.6%) of GAS6^High^ patients also had AXL^High^ co-expression (GAS6^High^/AXL^High^) (*P* < 0.001) (Table 5). Moreover, the co-expression relationship between AXL and GAS6 was further confirmed by IHC assays in serial sections of lung cancer metastasis tissues.

Univariate Cox model analysis indicated that GAS6 ^High^ and AXL^High^ tumoral expression levels were significantly associated with shorter OS (*P* < 0.01) (Tables 3, 6). We further evaluated the potential prognostic value of AXL expression coupled with GAS6 co-expression levels for overall survival of NSCLC metastasis patients. According to AXL/GAS6 co-expression pattern, the cases were divided into four groups: Group I with AXL^Low^ and GAS6^Low^; Group II with AXL^High^ and GAS6^Low^, and Group III with AXL^Low^ and GAS6^High^; and Group IV with AXL^High^ and GAS6^High^. Comparing to all the other groups, in multivariate Cox model analysis, the group IV (GAS6^High^/AXL^High^) patients displayed the worst survival prognosis correlation, robustly predicting poor outcomes both in the overall NSCLC metastasis cohort group (*P* = 0.001) (Table 4; Fig. 2f, Figure S2, i) and also in the NSCLC BM cohort group (Table 7; Fig. 3f, Figure S2, q) (*P* = 0.001).

## Discussion

AXL, a transforming protooncogene, was originally isolated from chronic myelogenous leukemia cells (O’Bryan et al. 1991). AXL has been reported to be ectopically expressed or overexpressed in a multitude of human cancers. GAS6 can bind to the extracellular domain of AXL leading to autophosphorylation of tyrosine residues on the intracellular tyrosine kinase domain of AXL, and induce activation of PI3K/AKT and MAPK/ ERK signaling pathways (Wu et al. 2014b). To the best of our knowledge, there are no reports to this date on the role of AXL–GAS6 in NSCLC BM, and the prognostic significance of AXL–GAS6 expression in lung cancer brain metastases remains undefined.

The epithelial–mesenchymal transition (EMT) process allows epithelial cells to lose their cell polarity and adopt mesenchymal-like phenotypes, thereby gaining the ability to migrate and invade surrounding tissue. EMT is correlated with the development of acquired resistance to chemotherapy (Fischer et al. 2015) and targeted therapy in a number of cancers (Shintani et al. 2016; Zhang et al. 2012). Previous studies reported by our group demonstrated that acquired epidermal growth factor receptor (EGFR) tyrosine kinase inhibitor (TKI)-resistant EGFR-mutant NSCLC cells not only overexpressed AXL, but also demonstrated a concomitant EMT-associated transcriptional program involving upregulation of vimentin (Zhang et al. 2012). Our recent studies also supported the notion that early adaptive precision drug-resistant escape in EGFR-mutant NSCLC under EGFR inhibitor treatment is associated with a quiescence cell state under transcriptomic-metabolomic cellular reprogramming that has an enhanced EMT-ness, cancer stemness, and upregulated vimentin (Thiagarajan et al. 2016). The EMT signature and AXL might be predictive biomarkers of drug response-resistance profile and therapeutic targets in patients with NSCLC (Fischer et al. 2015; Thiagarajan et al. 2016; Wu et al. 2014a). As overexpression of AXL/GAS6 can contribute to tumor invasion, metastasis especially to the brain, and drug resistance against chemotherapy and targeted therapies in NSCLC, the AXL/GAS6 pathway can be promising therapeutic target for clinical inhibition. There are a number of ongoing cancer clinical trials aiming at inhibiting the activity of AXL kinase, such as using the novel small-molecule inhibitor R428 (BGB324) to potently block autophosphorylation of AXL (Wu et al. 2014b). Final clinical study results upon completion of the trials would provide insights into the potential significance of AXL inhibition in NSCLC and other human cancers. Also, novel GAS6- and AXL-targeting inhibitors would be of value to be developed as well as combination inhibitory strategy co-targeting the two molecules.

NSCLC BM patients often have poor prognosis, with reported median overall survival time being only 7 months (Sperduto et al. 2012; Zimmermann et al. 2014). The median survival time of patients with metastatic NSCLC to the brain with tumoral EGFR mutations is more favorable at the range of 19–26 months, likely as a reflection of the favorable predictive and prognostic indices for EGFR mutations per se (Jiang et al. 2016; Luo et al. 2014; Welsh et al. 2013). In our current study, the median BM-OS time for the AXL^High^ group was 21 months and that for AXL^Low^ was 48 months. We did not include EGFR and ALK genotype information in our study analysis; and there was no complete treatment information on all the study patients. While EGFR and ALK kinase inhibitors as standard-of-care first-line targeted therapy for EGFR-mutated/ALK-rearranged NSCLC patients began in 2004 and 2011, respectively, in the US, for China, the genotype-guided therapy started since 2006 and 2013, respectively (Cohen et al. 2005; Sasaki and Janne 2011). The tumor tissues included in our current study were collected from December 2000 onwards. Moreover, not all the EGFR-mutated/ALK-rearranged patients would have received targeted therapy during the study period in China. Furthermore, AXL^High^ and GAS6^High^ expressions are each an independent predictor for poor OS in advanced lung cancer especially in the BM patients, but not in the subgroup of patients with metastasis to other organs. In addition, AXL^High^ expression level maintained its prognostic value in predicting shorter OS in N 2/3 stage, but not in N 0/1 stage. Of note, the metastatic brain tissues from lung primary was significantly associated with high co-expression of AXL/GAS6. AXL/GAS6 are known to be a pivotal signaling axis involved in EMT, which could conceivably be the underlying factor in leading to tumor progression and adverse prognosis in lung cancer BM. Our data support the finding that significant OS advantages were observed for the NSCLC BM in lung tissue subgroup compared with the brain tissue subgroup. We believe that there were confounding clinical factors in the NSCLC BM brain tissue subgroups inherent in the subgroup patients’ clinical conditions and scenarios as the basis of the decision of brain metastatic tissue craniotomy resection may contribute to the unfavorable outcome in this subgroup relative to the NSCLC BM lung tissue subgroup. These include factors such as symptomatic brain metastasis, and larger metastatic tumor size not amenable for gamma-knife radiosurgery in the brain tissue subgroup. In turn, these factors may also be correlated with the AXL–GAS6 expression as well. Moreover, the added potential perioperative risks may also contribute negatively to the brain tissue NSCLC BM subgroup patients’ survival outcome.

Based on our study results, we nominate GAS6^High^/AXL^High^ as a potential molecular determinant in NSCLC patients more likely to form metastatic sites at the CNS brain compartment, and is associated with poor survival outcome prognosis. Further preclinical translational model system to test and validate this hypothesis would be of great value. Our laboratory is pursuing these studies further to advance the clinical–translational impact of our study results.

Our current study has several limitations. First, it is a retrospective study with its intrinsic associated limitations. Second, our cohort size is modest; although it consists of well-annotated and unique sample cohort set. Third, there can be inherent methodological limitations and variations in the performance and scoring of standard IHC study and results. To minimize bias and methodological variations, we have herein adopted rigorous standardized assay methods in our study and the results were scored by two blinded independent well-trained clinic pathologists. Furthermore, additional studies with larger clinical sample cohort size from different centers would be of value to further validate our results.

Our results reveal that AXL–GAS6 signal axis potentially has a key role in NSCLC tumor progression and survival prognosis; and AXL alone or in combination with GAS6 may serve as feasible biomarker for prognostic prediction in patients with metastatic NSCLC to the brain. Combined co-expression analysis of AXL with GAS6 may serve to identify the high-risk NSCLC BM patients, and could engender an attractive therapeutic approach to prevent or combat brain metastases in NSCLC in the future.

## Electronic supplementary material

Below is the link to the electronic supplementary material.

Supplementary material 1 (DOCX 14 KB)

Supplementary material 2 (TIF 11173 KB)

Supplementary material 3 (TIF 64972 KB)

Supplementary material 4 (TIF 15948 KB)

## References

[CR1] Baek MY et al (2016) Epidermal growth factor receptor mutation and pattern of brain metastasis in patients with non-small cell lung cancer. Korean J Intern Med. doi:10.3904/kjim.2015.158 10.3904/kjim.2015.158 PMC5768530 27093978

[CR2] Barnholtz-Sloan JS, Sloan AE, Davis FG, Vigneau FD, Lai P, Sawaya RE (2004) Incidence proportions of brain metastases in patients diagnosed (1973 to 2001) in the Metropolitan Detroit Cancer Surveillance System. J Clin Oncol 22:2865–2872. doi:10.1200/JCO.2004.12.149 15254054 10.1200/JCO.2004.12.149

[CR3] Chen W et al (2016) Cancer statistics in China, 2015. CA Cancer J Clin 66:115–132. doi:10.3322/caac.21338 26808342 10.3322/caac.21338

[CR4] Cohen MH, Johnson JR, Chen YF, Sridhara R, Pazdur R (2005) FDA drug approval summary: erlotinib (Tarceva) tablets. Oncologist 10:461–466. doi:10.1634/theoncologist.10-7-461 16079312 10.1634/theoncologist.10-7-461

[CR5] Craven RJ et al (1995) Receptor tyrosine kinases expressed in metastatic colon cancer. Int J Cancer 60:791–797 7896447 10.1002/ijc.2910600611

[CR6] Ettinger DS et al (2012) Non-small cell lung cancer. J Natl Compr Canc Netw 10:1236–1271 23054877 10.6004/jnccn.2012.0130

[CR7] Fischer KR et al (2015) Epithelial-to-mesenchymal transition is not required for lung metastasis but contributes to chemoresistance. Nature 527:472–476 26560033 10.1038/nature15748 PMC4662610

[CR8] Hasanbasic I, Cuerquis J, Varnum B, Blostein MD (2004) Intracellular signaling pathways involved in Gas6–Axl-mediated survival of endothelial cells. Am J Physiol Heart Circ Physiol 287:H1207–H1213. doi:10.1152/ajpheart.00020.2004 15130893 10.1152/ajpheart.00020.2004

[CR9] He L, Zhang J, Jiang L, Jin C, Zhao Y, Yang G, Jia L (2010) Differential expression of Axl in hepatocellular carcinoma and correlation with tumor lymphatic metastasis. Mol Carcinog 49:882–891. doi:10.1002/mc.20664 20635370 10.1002/mc.20664

[CR10] Hector A et al (2010) The Axl receptor tyrosine kinase is an adverse prognostic factor and a therapeutic target in esophageal adenocarcinoma. Cancer Biol Ther 10:1009–1018. doi:10.4161/cbt.10.10.13248 20818175 10.4161/cbt.10.10.13248 PMC3025816

[CR11] Hong CC et al (2008) Receptor tyrosine kinase AXL is induced by chemotherapy drugs and overexpression of AXL confers drug resistance in acute myeloid leukemia. Cancer Lett 268:314–324. doi:10.1016/j.canlet.2008.04.017 18502572 10.1016/j.canlet.2008.04.017

[CR12] Ishikawa M et al (2013) Higher expression of receptor tyrosine kinase Axl, and differential expression of its ligand, Gas6, predict poor survival in lung adenocarcinoma patients. Ann Surg Oncol 20(Suppl 3):S467–S476. doi:10.1245/s10434-012-2795-3 23242819 10.1245/s10434-012-2795-3 PMC3853411

[CR13] Jiang T et al (2016) EGFR TKIs plus WBRT demonstrated no survival benefit other than that of TKIs alone in patients with NSCLC and EGFR mutation and brain metastases. J Thorac Oncol 11:1718–1728. doi:10.1016/j.jtho.2016.05.013 27237825 10.1016/j.jtho.2016.05.013

[CR14] Lee WP, Wen Y, Varnum B, Hung MC (2002) Akt is required for Axl–Gas6 signaling to protect cells from E1A-mediated apoptosis. Oncogene 21:329–336. doi:10.1038/sj.onc.1205066 11821945 10.1038/sj.onc.1205066

[CR15] Linger RM, Keating AK, Earp HS, Graham DK (2008) TAM receptor tyrosine kinases: biologic functions, signaling, and potential therapeutic targeting in human cancer. Adv Cancer Res 100:35–83. doi:10.1016/S0065-230X(08)00002-X 18620092 10.1016/S0065-230X(08)00002-X PMC3133732

[CR16] Luo D et al (2014) EGFR mutation status and its impact on survival of Chinese non-small cell lung cancer patients with brain metastases. Tumour Biol 35:2437–2444. doi:10.1007/s13277-013-1323-9 24197981 10.1007/s13277-013-1323-9

[CR17] Mujoomdar A, Austin JH, Malhotra R, Powell CA, Pearson GD, Shiau MC, Raftopoulos H (2007) Clinical predictors of metastatic disease to the brain from non-small cell lung carcinoma: primary tumor size, cell type, and lymph node metastases. Radiology 242:882–888. doi:10.1148/radiol.2423051707 17229875 10.1148/radiol.2423051707

[CR18] O’Bryan JP et al (1991) axl, a transforming gene isolated from primary human myeloid leukemia cells, encodes a novel receptor tyrosine kinase. Mol Cell Biol 11:5016–5031 1656220 10.1128/mcb.11.10.5016-5031.1991 PMC361494

[CR19] Sasaki T, Janne PA (2011) New strategies for treatment of ALK-rearranged non-small cell lung cancers. Clin Cancer Res 17:7213–7218. doi:10.1158/1078-0432.CCR-11-1404 22010214 10.1158/1078-0432.CCR-11-1404 PMC3477548

[CR20] Shieh YS, Lai CY, Kao YR, Shiah SG, Chu YW, Lee HS, Wu CW (2005) Expression of axl in lung adenocarcinoma and correlation with tumor progression. Neoplasia 7:1058–1064 16354588 10.1593/neo.05640 PMC1501169

[CR21] Shintani Y, Fujiwara A, Kimura T, Kawamura T, Funaki S, Minami M, Okumura M (2016) IL-6 secreted from cancer-associated fibroblasts mediates chemoresistance in NSCLC by increasing epithelial-mesenchymal transition signaling. J Thorac Oncol 11:1482–1492. doi:10.1016/j.jtho.2016.05.025 27287412 10.1016/j.jtho.2016.05.025

[CR22] Siegel RL, Miller KD, Jemal A (2016) Cancer statistics, 2016. CA Cancer J Clin 66:7–30. doi:10.3322/caac.21332 26742998 10.3322/caac.21332

[CR23] Sperduto PW et al (2010) Diagnosis-specific prognostic factors, indexes, and treatment outcomes for patients with newly diagnosed brain metastases: a multi-institutional analysis of 4,259 patients. Int J Radiat Oncol Biol Phys 77:655–661. doi:10.1016/j.ijrobp.2009.08.025 19942357 10.1016/j.ijrobp.2009.08.025

[CR24] Sperduto PW et al (2012) Summary report on the graded prognostic assessment: an accurate and facile diagnosis-specific tool to estimate survival for patients with brain metastases. J Clin Oncol 30:419–425. doi:10.1200/JCO.2011.38.0527 22203767 10.1200/JCO.2011.38.0527 PMC3269967

[CR25] Thiagarajan PS et al (2016) Transcriptomic-metabolomic reprogramming in EGFR-mutant NSCLC early adaptive drug escape linking TGFbeta2-bioenergetics-mitochondrial priming. Oncotarget 7:82013–82027. doi:10.18632/oncotarget.13307 27852038 10.18632/oncotarget.13307 PMC5347670

[CR26] Vaughan CA et al (2012) Gain-of-function activity of mutant p53 in lung cancer through up-regulation of receptor protein tyrosine kinase Axl genes. Cancer 3:491–502. doi:10.1177/1947601912462719 10.1177/1947601912462719 PMC3527987 23264849

[CR27] Welsh JW et al (2013) Phase II trial of erlotinib plus concurrent whole-brain radiation therapy for patients with brain metastases from non-small-cell lung cancer. J Clin Oncol 31:895–902. doi:10.1200/JCO.2011.40.1174 23341526 10.1200/JCO.2011.40.1174 PMC3577951

[CR28] Wimmel A, Glitz D, Kraus A, Roeder J, Schuermann M (2001) Axl receptor tyrosine kinase expression in human lung cancer cell lines correlates with cellular adhesion. Eur J Cancer 37:2264–2274 11677117 10.1016/s0959-8049(01)00271-4

[CR29] Wu F, Li J, Jang C, Wang J, Xiong J (2014a) The role of Axl in drug resistance and epithelial-to-mesenchymal transition of non-small cell lung carcinoma. Int J Clin Exp Pathol 7:6653–6661 25400744 PMC4230140

[CR30] Wu X, Liu X, Koul S, Lee CY, Zhang Z, Halmos B (2014b) AXL kinase as a novel target for cancer therapy. Oncotarget 5:9546–9563. doi:10.18632/oncotarget.2542 25337673 10.18632/oncotarget.2542 PMC4259419

[CR31] Zhang YX et al (2008) AXL is a potential target for therapeutic intervention in breast cancer progression. Cancer Res 68:1905–1915. doi:10.1158/0008-5472.CAN-07-2661 18339872 10.1158/0008-5472.CAN-07-2661

[CR32] Zhang Z et al (2012) Activation of the AXL kinase causes resistance to EGFR-targeted therapy in lung cancer. Nat Genet 44:852–860. doi:10.1038/ng.2330 22751098 10.1038/ng.2330 PMC3408577

[CR33] Zimmermann S, Dziadziuszko R, Peters S (2014) Indications and limitations of chemotherapy and targeted agents in non-small cell lung cancer brain metastases. Cancer Treat Rev 40:716–722. doi:10.1016/j.ctrv.2014.03.005 24759599 10.1016/j.ctrv.2014.03.005

