# Supplementary material for: AXL–GAS6 expression can predict for adverse prognosis in non-small cell lung cancer with brain metastases
Source: J Cancer Res Clin Oncol. 2017 May 27;143(10):1947–57. doi: 10.1007/s00432-017-2408-4 (PMC5599460; doi:10.1007/s00432-017-2408-4)
Supplement: Supplementary file 1 — Supplementary material 1 (DOCX 14 KB) [file 432_2017_2408_MOESM1_ESM.docx]

Supplementary Figure Legends

Figure S1. Spearman’s correlation analysis between AXL and GAS6 expression in 96 NSCLC metastases patients. The association between AXL and GAS6 expressions by IHC was estimated using Spearman's correlation with the scatter-plot and fitted straight-line, and AXL was found to have a positive correlation with GAS6 expression. R=0.532, *P*<0.001.

Figure S2. The correlation of NSCLC Overall Survival (OS) with different clinicopathologic characteristics. Survival curves were generated using the Kaplan-Meier method, and differences between curves were estimated by the log-rank test. *a-h*, CEA, T stage, pathology, LDH, gender, smoking, age, and differentiation have no statistically significant correlation with OS. *i,* Combined analysis of AXL and GAS6 co-expression and its correlation with NSCLC metastasis Overall Survival. The association of AXL/GAS6 high co-expression with overall survival (log-rank *P*<0.05) is shown here. Group I with AXL^Low^ and GAS6^Low^ (n = 37); Group II with AXL^High^ and GAS6^Low^ (n = 12); Group III with AXL^Low^ and GAS6^High^ (n = 12); Group IV, AXL^High^ and GAS6^High^ (n = 37). *j-p,* NSCLC BM-Overall Survival correlation with different clinicopathologic characteristics. Gender, differentiation, age, CEA, LDH, T stage, and smoking status have no statistically significant correlation with BM-OS. *q,* Combined analysis of AXL and GAS6 expression on NSCLC metastasis Overall Survival. The association of AXL/GAS6 co-expression with overall survival (log-rank *P*<0.05) is shown here. Group I with AXL^Low^ and GAS6^Low^ (n = 37); Group II with AXL^High^ and GAS6^Low^ (n = 12); Group III with AXL^Low^ and GAS6^High^ (n = 12); Group IV, AXL^High^ and GAS6^High^ (n = 37).

Figure S3. The correlation between AXL expression with NSCLC OM-OS. AXL, GAS6, N stage all have no statistically significant correlation with OM-OS.
